# Traumatic Spinal Cord Injury: Review of the Literature

**DOI:** 10.3390/jcm14113649

**Published:** 2025-05-22

**Authors:** Lori Zarmer, Maaz Khan, Glenn Islat, Hanan Alameddin, Maria Massey, Rabail Chaudhry

**Affiliations:** 1 Department of Anesthesiology and Pain Medicine, College of Medicine, University of Arizona, Tucson, AZ 85721, USA; lorizarmer@arizona.edu (L.Z.); gislat@arizona.edu (G.I.); mamassey@arizona.edu (M.M.); 2 Department of General Surgery, Montefiore Medical Center, Albert Einstein College of Medicine, Bronx, NY 10467, USA; maakhan@montefiore.org

**Keywords:** spinal cord injury, SCI, ASIA score, trauma, spinal injury, neurological deficits

## Abstract

Traumatic spinal cord injury (tSCI) is a devastating neurological disorder with profound effects on physical, psychological, and mental abilities. tSCI affects all age groups, with a higher incidence in elderly patients. There are many causes of tSCI, with motor vehicle accidents (MVA) and falls being the most common. The pathophysiology of tSCI is quite complex and involves primary and secondary injury. The primary injury directly results from the mechanical forces that caused the injury. Secondary injury is caused by long-term changes caused by inflammation, immune changes, and the formation of free radicals. Numerous studies have explored various medical and surgical treatment options that help mitigate long-term damage caused by tSCI and help improve quality of life. Currently, there are no treatments for tSCI that can reverse spinal cord damage or fully restore motor and sensory functions. However, many pharmacological and non-pharmacological options are being studied in tSCI patients. This review will discuss the background, pathophysiology, and clinical presentation of tSCI while also providing a detailed analysis of the recent advancements in treatment options.

## 1. Introduction

Traumatic spinal cord injury (tSCI) is caused by an external physical insult that results in damage to the spinal cord, leading to impaired motor, sensory, or autonomic functions. tSCIs can have devastating effects on patients’ physical and emotional well-being, causing severe disease burdens in both developed and developing countries [1]. Various treatment guidelines have been published on tSCI, yet there is a lack of consensus about the overall management strategies and ways to improve patients’ quality of life (QoL) [2]. In recent years, preclinical research has focused on identifying new therapeutic techniques, including cell transplantation, neuroregenerative, neuromodulatory techniques, and the use of robotics. There have been difficulties in translating preclinical research to patients due to the heterogeneity of patients and injuries. In this review, we will provide a comprehensive overview of tSCI pathophysiology and management techniques.

## 2. Epidemiology and Complications

There are multiple factors that affect the epidemiology of tSCI, including age, sex, and socioeconomic status. In the most recent estimates, analysis of the US nationwide inpatient sample databases found that the incidence of tSCI has remained relatively stable since the 1970s, with the most recent annual incidence estimate of tSCI at 54 cases per 1 million people in the United States [3,4]. Moreover, tSCI’s incidence has decreased in younger populations (16–44 years old), while increasing in the elderly population [3]. Higher incidences are seen in males compared to females, with 79% of new tSCI cases arising in males [4].

Causes of tSCI were found to differ between age groups. In the most recent estimates [3], 68% of tSCIs in the elderly were due to falls, an increase from 18% in 1997. In the 16–24 and 25–44 age groups, motor vehicle accidents (MVAs) comprised 44% and 40% of tSCIs, respectively. Firearm injuries were the second leading cause of tSCI in the younger population (16–44 years old). In the general population, the level of tSCI injury was most commonly cervical (~50%), followed by thoracic (35.2%) and lumbar (11%) [5].

The consequences of tSCI on patients’ health, general well-being, and quality of life are profound. A study showed that the average length of stay for tSCI patients in an acute care unit is 19 days, and the average rehabilitation length of stay is 37 days. About 30% of people with tSCI will be re-hospitalized at some point with genitourinary and skin complications, increasing the average length of stay to 18 days [3]. Life expectancy has not improved since the 1980s and remains well below the life expectancies of people without tSCI. The highest mortality rates are seen in the first year after injury [5]. Causes of death vary based on injury severity, age, and socioeconomic factors. In one study, pulmonary emboli were the most common cause of death in the first year after injury [6]. Unintentional injuries and suicide were more common among younger people. However, suicide decreased significantly 10 years post-injury [6]. Respiratory conditions and sepsis due to infectious diseases were common regardless of age and injury severity [6].

From an economic standpoint, tSCI represents a significant burden on the healthcare system and society. Studies have shown that the annual direct medical costs of tSCI in the USA are between USD 40,000 and USD 185,000. In addition to the medical costs, tSCI is one of the main causes of long-term disability worldwide, with an average global employment rate of 38% [7].

## 3. Pathophysiology of Presentation and Shock

tSCI can be classified into complete and incomplete tSCI (Table 1). In complete tSCI, there is no preservation of function below the level of injury and no sacral sparing, while incomplete tSCI is more nuanced. Incomplete tSCI is categorized based on the degree of motor and sensory function preserved below the injury [8].

The American Spinal Injury Association (ASIA) Impairment Scale is a widely accepted and validated tool used to classify spinal cord injuries according to severity [9,10,11,12].

Discomplete SCI is an injury pattern that is not fully understood despite being an established phenomenon for decades. Discomplete SCI is typically defined as a clinically complete SCI with evidence of residual neurological influence below the level of injury [13]. An early study of 88 complete SCI patients found that 84% of subjects demonstrated persistent motor connections between the brain and the lower extremities, measurable with electromyography (EMG) [13]. Similarly, a case–control EMG study of 18 complete cervical SCI patients found activation of paralyzed upper extremity muscle in all participants [14]. Functional MRI (fMRI) studies of clinically complete SCI patients have also shown activation of the somatosensory cortex in response to stimuli [15,16]. It is difficult to determine the prevalence of discomplete SCI as studies are often limited by small sample sizes and a lack of standardization. One study of 23 chronic SCI patients found strong evidence of discomplete injury in 17% of participants, which increased to 39% when including strong and possible evidence, and further increased to 61% when additionally including subjective reports of sensation [17]. Currently, discomplete SCI is an academic designation, but there is active research into whether these residual connections can be targeted and strengthened. A case report of an AIS-A patient with complete paralysis demonstrated increased spinal-cord-motor-evoked potentials after 16 weeks of neurorehabilitation [18]. Further investigation is needed to fully characterize the spectrum of discomplete injury and the potential for therapeutic targeting.

Another factor that clinicians need to be aware of is the difference between spinal and neurogenic shock, particularly as it affects the initial neurological assessment used to define the severity of tSCI [11]. Spinal shock outlines a four-phase progression, starting with an initial stage of areflexia and culminating in a final stage characterized by the return of deep tendon reflexes. Hyperreflexia is defined as the temporary state of flaccid paralysis. This is usually followed by loss of all functions—motor, sensory, autonomic, and reflexive—below the level of injury [11,19]. Neurogenic shock is a form of distributive shock most relevant in injuries above the T6 level. It is caused by the sudden loss of autonomic, mainly sympathetic, control of the heart’s pacemaker, contributing to hemodynamic instability. It is typically characterized by hypotension, relative bradycardia, wide pulse pressure, and warm pink extremities [14]. The pathophysiology behind tSCI consists of two distinct phases: the primary and secondary phases. The primary injury phase is the result of the initial mechanical forces received by the spinal cord at the time of injury [15,16]. These mechanical forces cause direct structural damage to neuronal and vascular tissue, causing an acute disruption of cellular function [15,16,17]. Broadly speaking, the mechanism of primary injury can then be classified into four main mechanisms of injury: impact with persistent compression (most common via burst fractures), impact alone, distraction, and laceration/transection [15,16,17].

**Figure 1:**
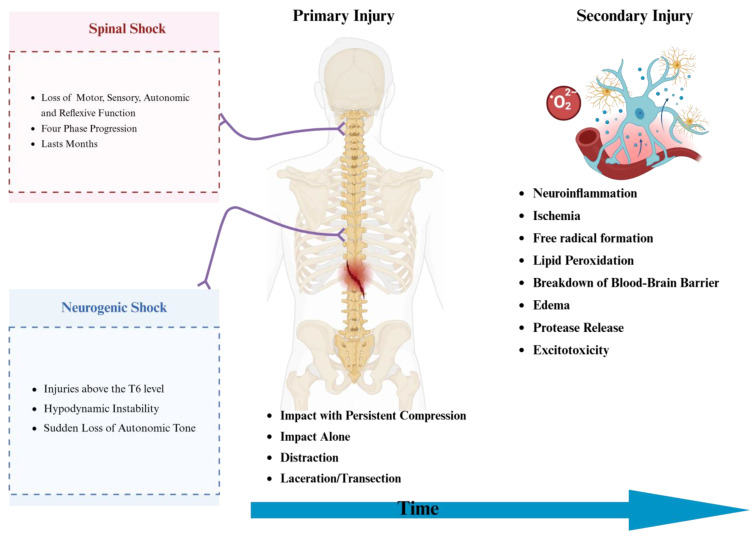
Illustration of tSCI Injury. Created in https://BioRender.com. Accessed on 15 April 2025.

The primary phase is short-lived and acute, while the secondary phase of injury continues for days to sometimes months [20,21,22]. The immune response plays an important role in propagating initial damage during the secondary phase. It does this through several mechanisms, such as neuroinflammation, ischemia, free radical formation, lipid peroxidation, breakdown of the blood–brain barrier, edema, protease release, and excitotoxicity [22]. The resulting inflammation can both exacerbate the initial injury and impair neural regeneration. Figure 1 illustrates the mechanisms of primary and secondary phases.

## 4. Evaluation

Evaluation of tSCI is based on an initial clinical assessment of the trauma, followed by imaging to further diagnose tSCI and determine treatment strategies. CT imaging is used to assess damage to the bony aspects of the spinal column, and MRI can be used to evaluate disc space and spinal cord involvement, as well as to evaluate for hematomas.

After appropriate imaging and clinical assessment, the injury can be classified using either the AOSpine spine injury classification system or the thoracolumbar injury classification system (TLICS) described in Table 2 [2,23]. AOSpine evaluates the injury in three ways: first, by morphology, generally via imaging; second, by neurologic status determined by the secondary survey; and last, by any patient-specific modifiers [24]. TLICS also uses morphology and neurologic status, but has a different third criterion, integrity of the posterior ligamentous complex [2]. Once the evaluation is complete, the patient’s stability, extent of other injuries from the trauma, ASIA injury assessment, injury classification, and imaging findings are used to guide medical and surgical management.

## 5. Medical Management

### 5.1. Role of Corticosteroids

The role of methylprednisolone in treating tSCI has long been debated. This debate arises from the assumption that corticosteroids prevent secondary damage after tSCI by preserving the spinal cord’s ultrastructure by reducing injury, decreasing free radical-catalyzed lipid peroxidation, enhancing impulse conduction and neuronal excitability, improving blood flow, reducing oxidative stress, and modulating the immune response [26]. Early data also suggested a potential benefit for steroid administration, but subsequent research, particularly the landmark National Acute Spinal Cord Injury Study [27,28,29], challenged this notion. These studies investigated the role of very high-dose methylprednisolone (bolus of methylprednisolone 30 mg/kg followed by an infusion of 5.4 mg/kg/h for 48 h) and indicated little benefit for their use. Although a secondary subgroup analysis revealed significant motor recovery in patients who received methylprednisolone within 8 h of injury, it also highlighted the dangers (including pneumonia and sepsis) of prolonged administration of high-dose steroids for 48 h. A more recent study conducted in 2023 with patients between 15 and 75 years of age failed to show clinical benefits of methylprednisolone use [30].

Due to a lack of level I and II evidence, the AANS/CNS guidelines from 2013 do not recommend the use of methylprednisolone for the treatment of tSCI [31]. However, AOSpine guidelines from 2017 recommend high-dose methylprednisolone for 24 h within 8 h of SCI in patients without medical contraindications [32].

### 5.2. Non-Steroidal Anti-Inflammatory Drugs (NSAIDs)

The benefit of NSAID administration in patients with tSCI has only been demonstrated in rat models, but there is a lack of evidence in clinical studies [33]. It is unclear whether the potential benefits outweigh the risks of the administration of NSAIDs after tSCI [34].

### 5.3. Monosialotetrahexosylganglioside (GM-1)

Glycosphingolipids, such as GM-1, are involved in several biological functions. GM-1 possesses the ability to activate tyrosine kinase inhibitors, promoting regeneration and enhanced neuronal plasticity [35]. An early phase II trial including 37 patients [36] and a subsequent phase III clinical trial of 760 patients [37] showed accelerated bladder, bowel, and motor recovery following treatment with GM-1 within the first three months; however, it revealed no significant benefit 26 weeks after injury. Furthermore, a Cochrane Systematic Review [38] described significant weaknesses within methodologies and hence concluded that there was no evidence to support its use in tSCI.

A recent retrospective analysis [39] from 2022 examined the effect of GM-1 on patients with severe tSCI and demonstrated a significantly higher total effective rate and significantly lower total incidence of complications. The study also observed significantly lower levels of IL-6, IL-8, TNF-α, and malondialdehyde (a marker of oxidative stress) compared to the control group [39].

### 5.4. Anti-CD11d Antibodies

Anti-CD11d antibodies represent a promising target for developing novel therapeutic options. The CD11/CD18 integrins on leukocytes bind to intercellular adhesion molecule-3 (ICAM-3) and vascular cell adhesion molecule-1 (VCAM-1) molecules, which become upregulated by damaged endothelial cells to promote leukocyte migration to injury sites. It is hypothesized that blocking this pathway with Anti-CD11d antibodies may offer neuroprotective benefits. Currently, this effect has only been demonstrated in rat models, but it warrants further investigation in human clinical trials.

### 5.5. Hepatocyte Growth Factor (HGF)

An exciting new development in potential therapeutics for tSCI is the use of HGF, which was shown to be a potent neurotrophic factor for the CNS. Recently, a double blind, randomized, multicenter phase I/II clinical trial [40] demonstrated the safety of treatment in patients who received intrathecal recombinant HGF during the acute stage of tSCI. A phase III clinical trial (ClinicalTrials.gov Identifier NCT04475224) is currently underway at five clinical sites in Japan. This study involves intrathecal injections of 0.6 mg HGF administered 72 h post-injury and repeated weekly for five weeks, with the final results awaiting release.

### 5.6. Macrophage Transplantation

Macrophages play a key role in tSCI’s pathophysiology. This role includes clearing tissue debris from sites of injury, modulating the immune system, and influencing spinal cord neurons, glial cells, and immune cells.

An early open-label phase I clinical trial assessed the treatment’s safety and tolerability and demonstrated improvement of the AIS score in three out of eight participants [41]. This followed a phase II clinical trial with 43 patients, which failed to show a significant difference between macrophage transplantation and placebo groups after six months [42]. Despite this, a Bayesian network meta-analysis revealed improved AIS scores and Functional Independence Measure (FIM) scores at 12 months [43], suggesting the need for additional studies.

### 5.7. Fibroblast Growth Factors (FGFs)

FGFs have shown efficacy in promoting axon regeneration, functional recovery, and neurotrophic effects. Two phase I/II clinical trials are currently investigating the use of FGFs in patients with tSCI. Both address the issue of crossing the blood–brain barrier and delivering FGFs to the CNS in their own unique way. The first one (ClinicalTrials.gov Identifier NCT02490501) is an open-label randomized control trial that uses a surgically implanted biodegradable device (SC0806) containing heparin-activated FGF-1 [44]; the results of this study have not been reported. The other is a double-blind RCT (ASCENT-ASCI study, ClinicalTrials.gov Identifier NCT01502631) [45] that uses SUN13837, a lipid-soluble injection containing FGF-1, in patients with acute SCI. The study concluded that while there was some evidence of minor neurological recovery of upper extremity motor function after 26 weeks, there was no significant clinical benefit [45].

### 5.8. Granulocyte Colony-Stimulating Factor (G-CSF)

G-CSF is a cytokine that can decrease both neuron and oligodendrocyte apoptosis, suppress inflammatory cytokines, and promote the proliferation/mobilization of neuronal cells. A recent meta-analysis compared the outcomes of six clinical trials involving G-CSF in incomplete tSCI and concluded that G-CSF for the treatment of incomplete tSCI may result in improved neurological outcomes when compared to the controls [25]. However, it is important to note that due to being limited by small sample sizes and heterogeneity between studies, more robust evidence is required to make an informed decision regarding G-CSF’s safety and efficacy.

### 5.9. Minocycline

Minocycline is a tetracycline antibiotic that has anti-inflammatory properties and shows promising neuroprotective capacity through multiple mechanisms. Minocycline has been shown to downregulate p38 mitogen-activated protein kinase (MAPK) and phospholipase A2 (PLA2) pro-inflammatory pathways, resulting in decreased apoptosis of oligodendrocytes [46,47]. It can also decrease oxidative stress and reactive oxygen species (ROS) and protect against glutamate toxicity [48,49]. It is also possible that minocycline exerts its neuroprotective effects by upregulating brain-derived neurotrophic factor (BDNF) and glial-derived neurotrophic factor (GDNF), particularly when combined with olfactory ensheathing cells [50]. There is currently a phase III clinical trial (Clinicaltrials.gov identifier NCT01828203) investigating whether intravenous minocycline administered twice daily, starting within 12 h of injury, might improve recovery of motor or sensory function in tSCI patients.

### 5.10. Chondroitinase ABC (ChABC) Enzyme

ChABC is a bacterial enzyme that degrades chondroitin sulphate proteoglycans (CSPGs), major constituents of the central nervous system’s extracellular matrix [51]. CSPGs are one of the components of the glial scar that inhibits regeneration. Administration of ChABC to patients with tSCI facilitates repair through multiple mechanisms, including axonal regeneration, enhanced connectivity of remaining pathways, and preservation of damaged neurons [52]. A randomized controlled study in canine models with chronic tSCI showed a 23% improvement in coordination in the ChABC group, with a subgroup of recipients regaining the ability to walk unassisted [53]. ChABC may also work synergistically with rehabilitation therapy, with co-administration resulting in recovery of function greater than either ChABC or rehabilitation alone [54].

### 5.11. Neuroimmunophilin Ligands

Neuroimmunophilin ligands, particularly FK-506 and cyclosporin A, are most widely recognized as mediators of immunosuppressants but have also been investigated for their nerve-regenerating potential [55,56]. The exact mechanism of these neuroprotective effects is a current area of research, although it has been proposed that the selective inhibition of FKBP51 is a fundamental component [57]. Studies have shown that FKBP51 promotes a glucocorticoid-based stress response that is beneficial to the central nervous system, with little or no impact on other glucocorticoid targets such as glucose metabolism and immunity [57]. GPI-1046, a synthetic derivative of FK506, has been shown to induce in vivo expression of the sodium-dependent glutamate transporter GLT1, ameliorating glutamate toxicity. An in vivo rodent model study that used a novel liposome to transport cyclosporin A across the brain–blood barrier showed significant improvement in motor function at both four weeks and three months following spinal cord injury [58].

### 5.12. Anti-Nogo-A Antibodies (ATI-355)

ATI-355 is a monoclonal antibody that targets the Nogo-A protein. Administration of ATI-355 to tSCI patients has been shown to significantly increase axonal sprouting and remyelination following an experimental autoimmune encephalomyelitis white matter lesioning study [59]. ATI-355 has also improved neurogenic lower urinary tract dysfunction following tSCI in rodent models, with improvement in maximum flow rate and voided volume [60]. A study of adult macaques with unilateral cervical lesions found that anti-Nogo-A antibodies resulted in increased axonal growth into scar tissue and across midline, suggesting a mechanism for circumventing scarred areas [61]. A phase I, open-label, multicenter study of ATI355 (ClinicalTrials.gov identifier: NCT00406016) in 48 patients with thoracic tSCI showed moderate improvements, with the greatest impacts seen in tetraplegic patients, of whom 11/19 had increased motor scores after 48 weeks [62]. A two-part phase I/phase II study of soluble Nogo-Receptor-Fc decoy AXER-204 (ClinicalTrails.gov identifier NCT03989440) in chronic tSCI patients showed no significant improvement in motor scores on initial analysis, with a post-hoc analysis revealing some improvement in patients with incomplete tSCI [63]. These early studies provide evidence that anti-Nogo antibodies could be beneficial for certain tSCI patients; however, there is a lack of consensus on whether ATI-355 administration results in any significant improvements in motor function [64,65].

### 5.13. Rho/ROCK Inhibitors (VX-210/Cethrin/BA-210, C3 Transferase, Fasudil, Y27632)

Rho is a GTPase protein that modulates a large variety of cellular functions through the downstream effector Rho-associated coiled-coil protein kinase (ROCK). Increased activity of this Rho/ROCK system is theorized to contribute to many neurological conditions, including tSCI. Rho is upregulated in gray matter, white matter, astrocytes, and oligodendrocytes after tSCI, further implicating Rho as a prominent player in the reaction to injury [66]. Inhibition of Rho/ROCK is under investigation as a method for counteracting the sequelae of secondary injury. One study showed that priming transplanted human fetal spinal-cord-derived neural precursor cells with the Rho/ROCK inhibitor fasudil promoted migration of grafted cells from the dorsal to the ventral spinal cord, although it did not demonstrate any significant impact on neuronal survival [67]. The treatment was also associated with modest increases in activated neurons as well as both GABAergic and glutamatergic interneurons at the injury site, highlighting the possible regulatory capacity of Rho/ROCK inhibitors [67]. Further evidence of this modulatory function is that the application of the Rho kinase inhibitor Y27632 induces morphological changes in astrocytes from the reactive A1 phenotype to the neuroprotective A2 phenotype, with associated enhanced recovery of motor capabilities [68].

A phase I/IIa open-label study of BA-210, trademarked as Cethrin, was able to demonstrate tolerability and safety in acute SCI patients, with a promising motor improvement observed in 31% of cervical SCI patients [69]. These initially exciting results were followed by a phase IIb/III study of VX-210, previously called BA-210 (ClinicalTrial.gov identifier: NCT02669849), which was stopped prematurely after analysis of the first 33% of patients who had completed the 6-month follow-up visit met the predefined futility rule [70]. These early trials did not show remarkable success, but the preclinical evidence of the regulatory actions of Rho/ROCK inhibitors may warrant future revisitation of these drugs as adjunct therapies.

### 5.14. B-Cell Depletion Therapies

B-cell depletion is another potential method of modulating the immune response to tSCI. Studies have shown that B-cell generation decreases following tSCI, possibly due to the action of endogenous glucocorticoids and norepinephrine cell depletion. This beneficial adaptation is believed to promote neurological recovery in tSCI patients. For instance, one study showed that transgenic mice lacking mature B-cells and T-cells experienced significantly improved axon regrowth and motor recovery six weeks after injury [71]. However, the use of B-cell depletion therapies to treat tSCI still requires further investigation [72,73,74].

### 5.15. Riluzole

Riluzole is a benzothiazole drug approved to treat amyotrophic lateral sclerosis by reducing glutamate toxicity [75]. Riluzole administration in tSCI models has been shown to reduce signal latency and increase evoked potential amplitude from spinal cord neurons after six weeks [76]. The most potent impacts on locomotor scores are seen when riluzole is given prior to spinal injury, with longer duration of pre-treatment being associated with greater improvements [77]. Although it is not realistic to anticipate and treat an injury preemptively, this highlights the importance of dosage and timing to achieve ideal concentrations in the spinal cord as soon as possible. These findings are supported by a systematic review of preclinical studies on traumatic and nontraumatic tSCI that demonstrated that riluzole results in improved locomotor, gait, and neuropathic pain outcomes [78].

A prospective, multicenter phase I trial (ClinicalTrials.gov identifier NCT00876889) involving 36 tSCI patients was able to demonstrate safety with no serious adverse effects, as well as increased motor recovery in patients with cervical spine injuries [79]. This was followed by an international, multicenter, prospective, randomized, and double-blinded phase II/III trial (ClinicalTrials.gov identifier NCT01597518) that did not show statistical significance in the primary outcome of upper extremity motor scores at 190 days, but was able to demonstrate significant clinical improvement in patients with incomplete injury [80]. Of note, this study was heavily impacted by the COVID-19 pandemic, as enrollment was suspended in May 2020 at 193 participants (55% of the intended enrollment), and the study was halted in April 2021 due to feasibility and safety concerns [80].

### 5.16. Stem Cell Therapy for tSCI

Stem cells are multipotent cells that can differentiate into a range of specialized cell types, which means they have the potential to replace lost cells in healing tissues. Stem cell transplantation is being investigated to combat the inevitable loss of healthy cells after tSCI. Induced pluripotent stem cells (iPSCs) are created by using transduction to reprogram certain genes in mature cells, typically fibroblasts, to revert them to an embryonic-like state [81]. Once cells are induced into pluripotency, they can be differentiated into various stem cell lineages. Neural stem cells (NSCs) directly replace lost neural cells, produce beneficial immunomodulatory effects, and form synapses after fully differentiating, resulting in improved motor function [82]. A phase I study (ClinicalTrials.gov Identifier: NCT01772810) demonstrated the safety of using human-spinal-cord-derived NSCs in a small cohort of patients; the study also noted that a few patients experienced functional improvement, indicating the promise of this therapy [83]. Additional studies have re-demonstrated the safety of NSCs without producing strong evidence for functional improvement, with a phase II study being terminated prematurely by the sponsor due to not achieving the set efficacy threshold [84,85].

Oligodendrocyte progenitor cells (OPCs) can be created from iPSCs and have been shown to integrate successfully into host tissues following contusive tSCI, with a subsequent increase in myelination and decrease in glial scarring [86]. A phase I/IIa multicenter trial (ClinicalTrials.gov Identifier NCT02302157) investigated the effect of administering increasing doses of OPCs and found that nearly all participants regained one level of neurological function after one year [87].

Mesenchymal stem cells (MSCs) are pluripotent stem cells obtained from a variety of sources, such as bone, umbilical cord, skeletal muscle, and dental pulp [81,88,89]. The phase I CELLTOP Clinical Trial (ClinicalTrials.gov Identifier NCT03308565) was able to demonstrate the safety of adipose-tissue derived MSCs (AD-MSCs) in ten SCI patients with no serious adverse events, with seven of the ten patients showing improvement in AIS grade after 96 weeks [90]. A phase I/II controlled single-blind clinical trial (Clinicaltrials.gov identifier NCT00816803) found that autologous adherent bone marrow cells combined with physical therapy significantly increased the number of patients who had ASIA motor score improvement of ten or greater points at the 18-month mark [91]. Another study used autologous bone marrow mesenchymal stem cells in chronic tSCI patients and demonstrated improvement in motor, sensory, and bladder functions not observed in the control group [92]. MSCs isolated from the umbilical cord are one of the lineages with the most robust evidence. Intrathecal injection of expanded Wharton jelly mesenchymal stromal cells (WJ-MSCs) was shown to result in significantly improved sensation in a randomized, double-blind, crossover, placebo-controlled, phase I/IIa clinical trial (NCT03003364) [93]. A randomized controlled trial (ClinicalTrials.gov Identifier NCT01393977) showed that treatment with umbilical cord MSCs (UCMSCs) results in improved neurological recovery of function compared to rehabilitation alone [94].

Due to the potential demonstrated in these early clinical trials, stem cells are a favored therapy that is expected to eventually enter clinical practice. A systematic review of 22 studies of MSC therapy for tSCIs, including 21 clinical trials, found that 12 studies demonstrated a change in AIS grade, 15 reported increases in the ASIA sensory score, 14 showed increases in the ASIA motor score, and nine studies demonstrated a change in the neurophysiological assessment [95]. The consensus supports the ability of MSCs to improve outcomes in tSCI patients, although the authors also note that many of the included studies had poor methodology or lacked control groups [95].

### 5.17. Extracellular Vesicle Therapy for tSCI

Extracellular vesicles (EVs), also called exosomes, are endosomes holding a variety of proteins, lipids, and other factors that allow targeted drug delivery of multiple components of chemically diverse factors to the spinal cord, minimizing drug side effects [81]. EVs are being investigated as a method to deliver RNAs to lesions in tSCI [96]. EVs loaded with small interfering RNAs administered intranasally to animal models were shown to be able to access the spinal cord and attenuate phosphatase and tensin homolog (PTEN) expression locally [97]. In another study, the administration of EVs enriched with microRNA-709 (miRNA-709) to mimic endogenous regulatory T-cell (Treg) cell exosomes has been shown to decrease microglia pyroptosis and improve motor recovery [98].

EVs can also be used to deliver MSCs [99]. MSC EVs have been shown to modulate the immune response by influencing macrophage polarization, reducing reactive astrocytes, and secreting neuroprotective miRNAs [99]. Intranasally administered umbilical cord MSC-derived EVs have been demonstrated to target vascular endothelial cells at the injury site and promote blood–spinal cord barrier integrity with associated improvement of functional motor recovery [100]. Schwann cell-derived EVs express the protein MFG-E8 that modulates the SOCS3/STAT3 immune signaling pathway, resulting in anti-inflammatory macrophage polarization, reduced apoptosis, and improvement in motor scores [101].

A limitation of implanted cells is that it is impossible to avoid some level of cell death once they are placed in the host tissue. Hydrogels have been investigated as a biomaterial scaffolding to support the viability of implanted cells and limit post-implantation losses [96,102]. Bone marrow stem cell-derived EVs loaded onto electroconductive hydrogels decrease inflammation by favoring the M2 microglial phenotype [102]. They also influence the cellular makeup by selectively promoting differentiation of NSCs into neurons and oligodendrocytes while inhibiting astrocyte differentiation [102]. There is evidence that electroconductive hydrogels themselves carry some benefit, but the combination of hydrogel and EVs was associated with the most significant gains in motor function in animal models [102]. Hydrogel patches carrying Schwann cell-derived EVs and methylprednisolone have been shown to favor anti-inflammatory M2 macrophage polarization and reduce neuronal apoptosis [103]. This composite patch additionally enhanced the electrophysiological activity of neurons, with treatment groups showing greater functional recovery [103]. Even though EV therapy offers a high degree of flexibility and customizability, further research is warranted to determine the most beneficial directions to develop in the future [96,104].

## 6. Surgical Management

Surgery is indicated when there is significant cord compression that causes progressive neurological impairment or a fracture that is not amenable to or responding to closed reduction, such as an unstable vertebral fracture [105]. The most effective clinical treatment for tSCI has been proven to be surgical decompression. This surgery focuses on repairing any axonal injury, disruption of blood vessels, and disruption of cellular membranes, the primary phases of injury. The goal of the surgery is to decompress the spinal cord, preventing further compression and neurological deficit, as well as realigning the spinal column to restore spinal stability and patient mobilization.

Surgical decompression can be achieved through one of two ways: direct decompression or indirect decompression. Direct decompression involves the resection of impinging structures, such as bone, ligaments, and disc material [106]. This can be combined with a durotomy or duraplasty to restore the normal flow of cerebrospinal fluid, decreasing intraspinal pressure and alleviating compression on the dura mater [107].

Indirect decompression is a group of procedures that provide decompression effects without resecting tissue by creating distraction forces that can directly increase the size of the epidural space and reduce the disc height or indirectly reduce the fracture, such as in the ligamentotaxis technique [106]. Global spinal alignment procedures are another indirect decompression technique used to remove the posterior compressing force directly or with instrumentation. This allows the spinal cord to migrate dorsally and indirectly decompress from anterior compressing forces [106].

Regardless of the technique, early intervention is key as it can prevent further irreversible cell damage and death [108]. Recent studies have shown that decompression surgery within 12 h after a TSCI correlated with improved neurological outcomes and AIS score by at least two grades [2,11,26]. Patients in the AIS A and AIS B groups who received early decompression surgery experienced shorter hospital length of stay [11].

### 6.1. Spinal Cord Cooling

Therapeutic hypothermia for tSCI is not a new concept and has been studied experimentally and clinically for a variety of disorders, including cerebral aneurysm, traumatic brain injury (TBI), aortic arch aneurysm, cardiac arrest, and acute tSCI [109]. It is hypothesized that therapeutic hypothermia can inhibit several metabolic pathways, inflammatory responses, and apoptotic processes that occur following an ischemia cascade [110]. Several preclinical studies in animals have demonstrated the safety and efficacy of both systemic and local hypothermia for tSCI.

### 6.2. Functional Electrical Stimulation (FES)

FES is a means of recovery in the rehabilitation setting that aims to generate functional movements in paralyzed and damaged muscles by imitating neural signals that are sent to peripheral nerves below the level of the spinal cord lesion and enhancing oligodendrocyte and myelin formation [111,112,113]. Few studies evaluated the outcome of FES-assisted training of organ functions such as cardiovascular health and bladder function; however, FES use in tSCI patients needs to be further explored [114].

### 6.3. Spinal Cord Stimulation (SCS)

SCS involves implanting electrodes into the epidural space, which provides direct stimulation to spinal cord neurons [115]. It can aid in managing nerve pain by blocking ascending nociception with tonic or high-frequency impulses consistent with the “Gate control” theory of analgesia [115]. A developing field of interest is using SCS to potentiate activity-dependent neuroplasticity after SCI in order to promote recovery [116]. Current research from animal studies and case reports seems to indicate that SCS works synergistically with rehabilitative training to improve function [117]. SCS used during neurorehabilitation has been shown in an animal model to increase the activity of a specific subgroup of neurons necessary for recovery of walking ability [118].

There is active interest in adapting this technology from analgesia to the restoration of function by modifying the stimulation produced. Three individuals with chronic SCI regained voluntary walking, stride adjustment, and stopping after implantation of SCS devices delivering timed trains of stimulation to the lumbosacral spinal cord [119]. Another study of six SCI patients demonstrated that transcutaneous SCS results in recovery of upper extremity function, including fine motor control, with function maintained for at least three to six months after treatment [120]. These early results show promise for SCS as a modality for SCI patients to recover significant motor function, with larger-scale studies needed to determine the true scope of efficacy.

### 6.4. Cerebrospinal Fluid Drainage (CSFD)

CSFD [121] has been studied to lower intrathecal pressure (ITP) and improve spinal cord perfusion pressure (SCPP) in patients with tSCI [2,122].

There have been conflicting findings in the literature about the effectiveness of CSFD in lowering ITP. Animal studies have demonstrated that MAP elevation and CSFD combined significantly improved SCPP compared to either therapy individually [123]. An RCT of 22 acute tSCI patients found that CSFD increased the mean ITP postoperatively [124], while another prospective trial of 13 patients found no change in intraspinal pressure [125]. However, a 2023 study found that post-decompression intraoperative CSFD with concomitant MAP elevation led to a decline in mean ITP, with similar adverse effect rates compared to controls [122].

Larger sample sizes and longer follow-up periods are needed to ascertain the efficacy of CSFD as a treatment in tSCI [123]. However, CSFD is shown to be a promising intervention to reduce ITP and improve SCPP in tSCI.

### 6.5. Tissue Scaffolding

Collagen scaffold systems have demonstrated improved nerve regeneration and functional recovery in tSCI animal models [126]. Biodegradable synthetic polymer scaffolds have shown potential as a tSCI intervention in preclinical settings [127]. Furthermore, stem cell therapy in preclinical studies improved motor function through nerve cell proliferation and differentiation [126]. Recent research has focused on combination therapies involving scaffold systems loaded with stem cells. A study from 2017 reported improved nerve regeneration, myelination, and functional recovery in canine models [126], while in 2024, another group reported increased motor function recovery and tissue repair in rat models [128].

Collagen scaffolds loaded with human umbilical cord mesenchymal cells were implanted in two patients with complete tSCI. After one year, recovery of sensory and motor functions was observed in both patients, and no adverse effects attributed to the therapy were observed [129]. Another study has demonstrated the safety of tissue scaffolding implantation in a human subject [130]. Despite these successes, clinical applications of tissue scaffolding are still in development.

## 7. Bowel Management and Diet

Neurogenic bowel (NB) affects up to half of patients with tSCI [131] and results from the disruption of central nervous control, leading to significant challenges in bowel management and reduced quality of life.

Prospective survey studies show that tSCI patients experience higher rates of fecal incontinence [132], constipation [133], and abdominal issues compared to the general population, with neurogenic bowel dysfunction causing both physical and psychological impacts.

The management of bowel disorders and constipation in tSCI patients mainly involves evacuation schedules, diet, fluid intake, and digital stimulation [134]. However, the outcome highly varies among participants.

Other methods of managing bowel function in patients with tSCI include pharmacological prokinetic agents and stool softeners such as cisapride and metoclopramide [134]. For severe cases, surgical interventions such as colostomies and the Malone anterograde continence enema (MACE) procedure offer the best long-term outcomes (reduced complication rates, lower incidence of autonomic dysreflexia, and fitted with patient preferences), though future studies are required for further insights on surgical impacts [132,134,135,136].

## 8. Urological Considerations

Patients with spinal cord injury display involuntary reflux detrusor contractions during bladder filling (similar to detrusor overactivity), with reflex contraction of the distal sphincter resulting in detrusor-sphincter dyssynergia, increasing the risk of urinary tract infections, hydronephrosis, renal stones, and post-renal kidney failure [8,137]. Patients with spinal cord injury often suffer from permanent loss of independent voiding, leading to scheduled clean intermittent self-catheterization. Other options include external male urinary catheters or suprapubic catheters, which depend more on upper extremity function and/or degree of retention [8,137].

Micturition through straining and sacral root stimulation is an option for patients with some retained sensation and voluntary control, but these often prove to be inadequate. In the setting of bladder spasms, oxybutynin is helpful at providing patient comfort while also reducing incontinence associated with spasms. Tamsulosin may also be used as it reduces sphincter tone through smooth muscle relaxation, improving bladder emptying [138]. However, this should be avoided in the acute injury period, as alpha inhibitors may inadvertently lower the blood pressure and reduce spinal cord perfusion.

## 9. VTE Prophylaxis

Patients with acute tSCI are at higher risk of venous thromboembolism (VTE) [139]. Evidence-based guidelines from the American College of Chest Physicians recommend mechanical methods and pharmacological thromboprophylaxis for at least the first two weeks following the injury [140]. Pharmacological thromboprophylaxis is usually conducted with low-molecular-weight heparin (LMWH) within 72 h of injury, while mechanical methods involve the use of intermittent pneumatic compression devices [8,140]. Though the majority of DVT events occur within the first eight weeks of injury, we are unaware of any trials specifically addressing the duration of DVT prophylaxis. The Spinal Cord Consortium, however, recommends at least eight weeks of pharmacologic DVT prophylaxis following acute tSCI [141,142]. The routine use of IVC filters is not recommended, and multiple studies in the trauma literature were unable to establish its efficacy in reducing overall or PE-related mortality, with a recent study even finding prophylactic IVC filtration to be associated with an increased risk of VTE in adults with spinal cord injuries [143].

## 10. Mental Health

Often overlooked are the effects on mental health and perceived quality of life. Patients with tSCI have reported poorer QoL than those without disabilities [144,145]. One study found a high incidence of depression (37%), anxiety (30%), and post-traumatic stress disorder (8.4%) in patients with SCI, with nearly half (48.5%) of patients affected by some form of mental health disorder [146]. Currently, no systematic strategies exist to address this issue. Healthcare providers should remain vigilant about the heightened prevalence of psychiatric disorders and maintain a low threshold for connecting patients with appropriate mental health resources when necessary [8].

## 11. Mobility and Discharge Disposition

Physical rehabilitation is one of the most intricate aspects of post-acute care for patients with SCI, particularly those with complete injuries who experience a sudden loss of movement in their lower and possibly upper extremities [8]. An acute loss of mobility has cascading consequences, including an increased risk of diabetes, obesity, and pressure ulcers [147], making it essential to engage patients with acute spinal cord injury in physical therapy as early as post-injury day one, with a target of over 20 min of maximum-tolerated aerobic activity per day [148]. A higher quality of life for individuals with tSCI is closely tied to social reintegration, a complex process involving independence, productive behavior, and maintaining interpersonal relationships [144]. A study in Slovenia interviewed 62 SCI individuals (Th6–Th12 injury level), half of whom were physically active. Participants rated interpersonal relationships highest and material prosperity lowest. Those engaged in physical activity reported higher subjective quality of life across all areas, highlighting the potential benefits of sports participation for SCI individuals [144]. Additional aerobic activities, such as swimming, walking, and wheelchair mobility (as tolerated), are positively correlated with improved quality of life outcomes; however, these benefits have not yet been evaluated against no-activity in randomized controlled trials [149].

Consequently, timely discharge to specialized tSCI rehabilitation centers is imperative, as these facilities provide tailored physical therapy and resources for tSCI patients, with evidence suggesting that appropriate rehabilitation can significantly extend the lifespan of individuals with spinal cord injuries [150].

## 12. Summary of Recommendations

Traumatic spinal cord injury (tSCI) represents a complex interplay between physical trauma, the immune system, and the central nervous system. Despite the significant healthcare burden of tSCI, there has not been much advancement in terms of treatment options and outcomes over the past few decades. Surgical decompression remains the primary treatment option addressing initial injury, providing tangible benefits when appropriate. However, the majority of treatments under investigation target secondary injury, primarily related to the immune response following spinal cord lesions, such as stem cell therapy. While early trials have typically shown safety, there is no consensus on whether these treatments provide significant clinical benefits. Some promising therapies, such as glycosphingolipids, ChABCs, neuroimmunophilin ligands, and transplantation of MSCs, have evidence supporting their potential for clinical benefit. Many other treatments, including HGF, macrophage transplantation, and minocycline, require further study and characterization. Further exploration of treatments for tSCI is needed before clinical practice can change and novel ideas can be incorporated into standard rehabilitation interventions.

## Figures and Tables

**Table 1:** American Spinal Injury Association Impairment Scale.

A	Complete	No motor or sensory function remains in the sacral segments S4–S5.
B	Incomplete	Sensory function is retained below the neurological level, but motor function is absent, including the sacral segments S4–S5.
C	Incomplete	Motor function is present below the neurological level, but more than half of the key muscles have a grade lower than 3
D	Incomplete	Motor function is preserved below the neurological level, and at least half of the key muscles below the neurological level have a muscle grade of 3 or higher.
E	Normal	Motor and sensory function are normal.

**Table 2:** Medical management of tSCI.

Intervention	Research Stage	Findings and Outcomes
Steroids (methylprednisolone)	Clinical	There is no difference in neurological recovery of motor function or pinprick and light touch sensation.
Non-Steroidal Anti-inflammatory Drugs (NSAIDs)	Animal	Benefits only be observed in animal models. No clinical significance in patients.
Monosialotetrahexosylganglioside (GM-1)	Clinical	Conflicting results: some studies show improvement in tSCI signs and symptoms, while others fail to provide evidence of effectiveness.
Anti-CD11d Antibodies	Animal	Only studied in rat models
Fibroblast Growth Factors (FGFs)	Clinical	No significant clinical benefit
Macrophage Transplantation	Clinical	No significant difference between macrophage transplantation and placebo group.
Granulocyte Colony-Stimulating Factor (G-CSF)	Clinical	G-CSF for the treatment of incomplete tSCI may result in improved neurological outcomes compared to controls [25].
Minocycline	Clinical	No evidence of neurological improvement
Chondroitinase ABC (ChABC) Enzyme	Clinical	A randomized controlled study in canine models with chronic tSCI showed a 23% improvement in coordination in the ChABC group, with a subgroup of recipients regaining the ability to walk unassisted.
Neuroimmunophilin Ligands	Animal	In vivo rodent model study showed significant improvement in motor function at both four weeks and three months following spinal cord injury.
Anti-Nogo-A Antibodies (ATI-355)	Clinical	There is a lack of consensus on whether ATI-355 administration results in any significant motor improvements.
Rho/ROCK Inhibitors (VX-210/Cethrin/BA-210, C3 transferase, fasudil, Y27632)	Clinical	Studies did not show remarkable success, but preclinical evidence of the regulatory actions of Rho/ROCK inhibitors may warrant future revisitation of these drugs as adjunct therapies.
B-Cell Depletion Therapies	Animal	The use of B-cell depletion therapies to treat tSCI still requires further investigation.
Riluzole	Clinical	Studies did not show statistical significance in the primary outcome of upper extremity motor scores.
Stem Cell Therapy for tSCI	Clinical	There is no strong evidence to support functional improvement using neural stem cells (NSCs). Administering increasing doses of oligodendrocyte progenitor cells (OPCs) might help with one level of neurological function after one year. Mesenchymal stem cell (MSC) administration results in improved neurological recovery of function compared to rehabilitation alone.
Extracellular Vesicle Therapy for tSCI	Clinical	MSC therapy might increase the ASIA sensory score, 14 showed increases in the ASIA motor score, and improve neurophysiological assessment
